# Physiological Aldosterone Concentrations Are Associated with Alterations of Lipid Metabolism: Observations from the General Population

**DOI:** 10.1155/2018/4128174

**Published:** 2018-03-27

**Authors:** M. Hannich, H. Wallaschofski, M. Nauck, M. Reincke, C. Adolf, H. Völzke, R. Rettig, A. Hannemann

**Affiliations:** ^1^Institute of Clinical Chemistry and Laboratory Medicine, University Medicine Greifswald, Greifswald, Germany; ^2^German Centre for Cardiovascular Research, Partner site Greifswald, Greifswald, Germany; ^3^Medical Department IV, Klinikum Innenstadt, Ludwig-Maximilian University of Munich, Munich, Germany; ^4^Institute for Community Medicine, University Medicine Greifswald, Greifswald, Germany; ^5^Institute of Physiology, University Medicine Greifswald, Greifswald, Germany

## Abstract

**Objective:**

Aldosterone and high-density lipoprotein cholesterol (HDL-C) are involved in many pathophysiological processes that contribute to the development of cardiovascular diseases. Previously, associations between the concentrations of aldosterone and certain components of the lipid metabolism in the peripheral circulation were suggested, but data from the general population is sparse. We therefore aimed to assess the associations between aldosterone and HDL-C, low-density lipoprotein cholesterol (LDL-C), total cholesterol, triglycerides, or non-HDL-C in the general adult population.

**Methods:**

Data from 793 men and 938 women aged 25–85 years who participated in the first follow-up of the Study of Health in Pomerania were obtained. The associations of aldosterone with serum lipid concentrations were assessed in multivariable linear regression models adjusted for sex, age, body mass index (BMI), estimated glomerular filtration rate (eGFR), and HbA1c.

**Results:**

The linear regression models showed statistically significant positive associations of aldosterone with LDL-C (*β*-coefficient = 0.022, standard error = 0.010, *p* = 0.03) and non-HDL-C (*β*-coefficient = 0.023, standard error = 0.009, *p* = 0.01) as well as an inverse association of aldosterone with HDL-C (*β*-coefficient = −0.022, standard error = 0.011, *p* = 0.04).

**Conclusions:**

The present data show that plasma aldosterone is positively associated with LDL-C and non-HDL-C and inversely associated with HDL-C in the general population. Our data thus suggests that aldosterone concentrations within the physiological range may be related to alterations of lipid metabolism.

## 1. Introduction

Cardiovascular diseases are the leading cause of death worldwide [1]. Among other factors, high low-density lipoprotein cholesterol (LDL-C) and low high-density lipoprotein cholesterol (HDL-C) concentrations are important risk factors for cardiovascular disease [2].

Aldosterone, a hormone produced in the zona glomerulosa of the adrenal glands, has a major impact on metabolic pathways and is connected to multiple cardiovascular pathologies including endothelial dysfunction, thrombosis, and myocardial as well as vascular fibrosis [3]. Several studies [4–9] suggest that there may be associations between the concentrations of aldosterone and certain components of lipid metabolism in peripheral circulation. In an experimental setup including 30 volunteers, Goodfriend et al. [6] described a negative association between aldosterone and HDL-C and a positive correlation between aldosterone and triglycerides. Several other studies [5, 7–9] described associations between aldosterone and metabolic syndrome, which comprises elevated serum HDL-C and triglyceride concentrations. Thus, in 2292 Framingham Offspring Study participants, aldosterone was positively associated with incident metabolic syndrome and inversely with changes in HDL-C [8]. Furthermore, lipid metabolism deteriorated after successful treatment of primary aldosteronism [4].

While these studies [4–9] suggest that there are associations between aldosterone and the components of lipid metabolism in certain patient groups, data from the general population is sparse. We therefore aimed to assess the associations between aldosterone and HDL-C, LDL-C, total cholesterol, triglycerides, or non-HDL-C in the general adult population.

## 2. Subjects and Methods

### 2.1. Study Population

Data were obtained from the first follow-up of the longitudinal Study of Health in Pomerania (SHIP-1). SHIP is a population-based project in the northeast of Germany. From the population registration offices, a representative sample including 7008 adults aged 20 to 79 years was selected. Baseline examinations (SHIP-0) were conducted between 1997 and 2001 including 4308 subjects. Starting in March 2002, the first five-year follow-up examination (SHIP-1) was performed. SHIP-1 was finished in July 2006 and comprised 3300 participants, who received aldosterone measurements. Details on study design and sampling methods are given elsewhere [10]. The study participants provided information on sociodemographic characteristics, lifestyle, and medical histories. They further underwent standardized physical examinations, including measurements of body height and weight and provided blood samples. Body mass index (BMI) was calculated as weight (kg)/height^2^ (m^2^). Systolic and diastolic blood pressures were measured three times on the right arm of seated subjects, using a digital blood pressure monitor (HEM-705CP, Omron Corporation, Tokyo, Japan). The mean of the second and third measurements was used for statistical analyses. Hypertension was defined as systolic blood pressure ≥ 140 mmHg or diastolic blood pressure ≥ 90 mmHg or self-reported intake of antihypertensive mediation. Diabetes mellitus was defined when the participants reported a respective physician's diagnosis and intake of antidiabetic medication and had an HbA1c ≥ 6.5% or a random glucose concentration ≥ 11.1 mmol/l. The participants' medication was categorised according to the anatomical-therapeutic-chemical (ATC) classification code. The intake of statins was defined according to ATC code C10. The intake of antidiabetic medication was defined according to ATC code A10, oral contraceptives as ATC code G03A. The intake of medication that alters aldosterone or renin concentrations was defined as diuretics including aldosterone antagonists (ATC C03), beta blockers (ATC C07) or other antiadrenergic agents (ATC C02), calcium channel blockers (ATC C08), angiotensin converting enzyme inhibitors (ATC C09A-B), or angiotensin receptor blockers (ATC C09C-D). Of the 3300 participants, we excluded all those (overlap exists) with missing data on aldosterone or serum lipid concentrations (*n* = 36), missing data on BMI (*n* = 10) or HbA1c (*n* = 33), subjects with renal insufficiency (defined as estimated glomerular filtration rate (eGFR) < 30 ml/min/1.73 m^2^), or missing information on renal function (*n* = 32); with aldosterone or the aldosterone-to-renin ratio outside the study-specific reference range (*n* = 287) [11]; and with intake of statins or medication that alters aldosterone or renin concentrations (RAAS, *n* = 1408) and all pregnant women (*n* = 10). This resulted in a study population of 1731 subjects.

All participants gave written, informed consent. The study conformed to the principles of the Declaration of Helsinki and was approved by the Ethics Committee of the Board of Physicians Mecklenburg-West Pomerania at the University of Greifswald.

### 2.2. Laboratory Measurements

Nonfasting blood samples were taken from the cubital vein of participants in the supine position. Aldosterone and renin concentrations were measured in EDTA plasma by radioimmunometric procedures (aldosterone: Coat-A-Count Aldosterone, Siemens Healthcare Diagnostics, Eschborn, Germany; renin: Renin III generation, Cisbio Bioassay, Bagnols-sur-Cèze Cedex, France) as previously described [11]. The aldosterone-to-renin ratio was calculated by dividing aldosterone by renin concentrations. Sodium and potassium concentrations were determined in serum on the Dimension RxL (Siemens Healthcare Diagnostics, Eschborn, Germany). Serum lipid concentrations were quantified by lipoprotein electrophoresis (HDL-C and LDL-C: HELENA SAS-3 system; Helena 7 BioSciences Europe, Tyne and Wear, UK) or were measured on the Dimension RxL (Siemens Healthcare Diagnostics, Eschborn, Germany). Triglycerides were measured enzymatically and total cholesterol was determined by the CHOD-PAP method. Non-HDL-C was calculated as difference between total and HDL-C.

### 2.3. Statistical Analyses

General characteristics of the study population are expressed as medians with 1st–3rd quartiles (continuous data) or as proportions (categorical data) according to sex-specific quartiles of the aldosterone concentration. Group differences were tested for statistical significance with chi-squared or Kruskal-Wallis tests. The associations of aldosterone with serum lipid concentrations were assessed in multivariable linear regression models adjusted for sex, age, BMI, eGFR, and HbA1c. We further assessed the associations of the aldosterone-to-renin ratio with serum lipid concentrations to examine whether the associations are driven by aldosterone per se or by the relation of aldosterone-to-renin. For the regression analyses, we log transformed exposure and outcome variables to obtain normally distributed residuals. We report *β*-coefficients with standard errors and *p* values. A value of *p* < 0.05 was considered statistically significant. All statistical analyses were performed with SAS 9.4 (SAS Institute Inc., Cary, North Carolina, USA).

## 3. Results

The study population included 793 men and 938 women aged 25–86 years (median 47 years, 1st–3rd quartile 37–57 years). Subjects in the highest aldosterone quartile were on average four years younger than those in the lowest aldosterone quartile (42 versus 46 years, *p* < 0.01, Table 1). Further group differences in systolic or diastolic blood pressures, eGFR, HbA1c, or BMI were not statistically significant. Also, total cholesterol, LDL cholesterol, non-HDL cholesterol, and triglycerides were not significantly different in the aldosterone quartiles. In contrast, HDL-C decreased with increasing aldosterone. Thus, HDL-C was 1.22 mmol/l (1st–3rd quartile 0.95–1.55 mmol/l) in the lowest aldosterone quartile and 1.13 mmol/l (1st–3rd quartile 0.90–1.45 mmol/l) in the highest aldosterone quartile (*p* = 0.04).

**Figure 1:**
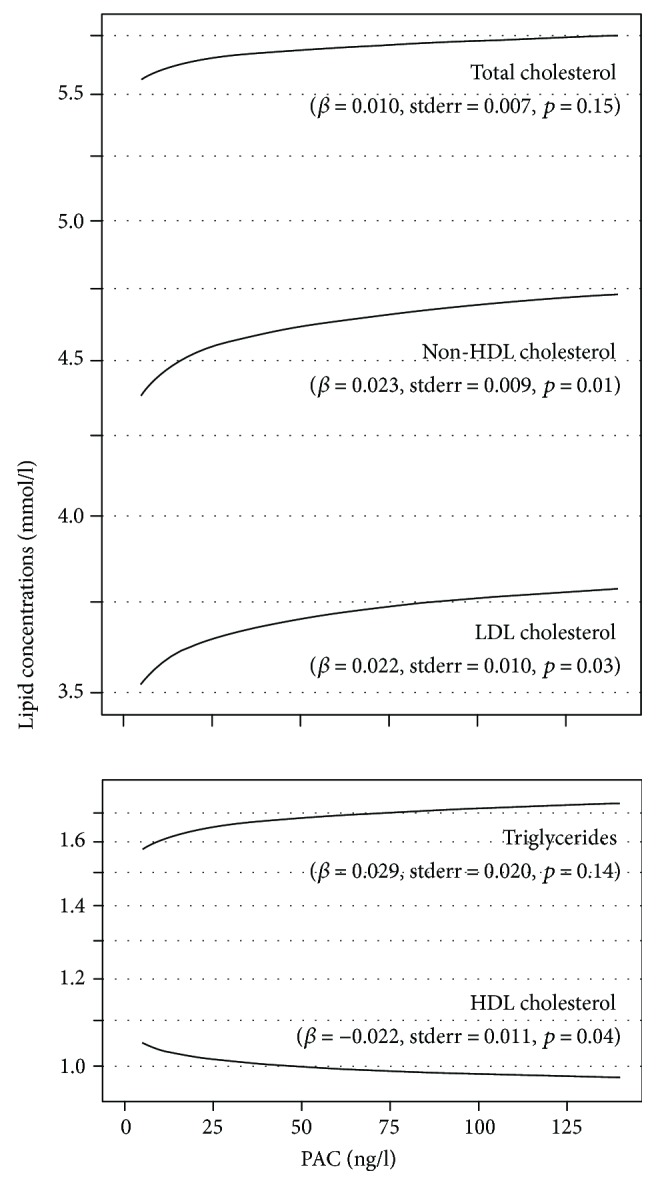
Associations between plasma aldosterone concentration (PAC) and serum lipid concentrations. Results from multivariable linear regression analyses adjusted for age, sex, body mass index, estimated glomerular filtration rate, and HbA1c. *β*-coefficients, standard errors (stderr), and *p* values are given. PAC and the serum lipid concentrations were log transformed before being entered in the regression models and back transformed for the illustration in the figure. The *y*-axis is log scaled.

The linear regression models (Figure 1) revealed positive associations of aldosterone with LDL-C (*β*-coefficient = 0.022, standard error = 0.010, *p* = 0.03) and non-HDL-C (*β*-coefficient = 0.023, standard error = 0.009, *p* = 0.01) as well as an inverse association of aldosterone with HDL-C (*β*-coefficient = −0.022, standard error = 0.011, *p* = 0.04), while the associations with total cholesterol (*β*-coefficient = 0.010, standard error = 0.007, *p* = 0.15) and triglycerides (*β*-coefficient = 0.029, standard error = 0.020, *p* = 0.14) were not statistically significant. The relations of the aldosterone-to-renin ratio with the lipid concentrations pointed in the same direction but none of the examined associations was statistically significant (total cholesterol: *β*-coefficient = 0.009, standard error = 0.006, *p* = 0.17; LDL-C: *β*-coefficient = 0.012, standard error = 0.009, *p* = 0.16; HDL-C: *β*-coefficient = −0.004, standard error = 0.010, *p* = 0.71; non-HDL-C: *β*-coefficient = 0.015, standard error = 0.009, *p* = 0.08; and triglycerides: *β*-coefficient = 0.019, standard error = 0.018, *p* = 0.30).

## 4. Discussion

The results of several cross-sectional [5, 7] and longitudinal [8, 9] studies that included patients with metabolic syndrome suggest that increased plasma aldosterone is associated with an altered lipid metabolism, including decreased HDL-C [5, 7–9] and increased triglycerides [7, 9]. Another study in 2891 Framingham Offspring Study participants [12] and without focus on the metabolic syndrome demonstrated an association between higher aldosterone and an elevated total/HDL-C ratio but not with HDL-C alone. Further lipid metabolism components were not evaluated in this study [12]. Our results confirm the previously reported inverse association of aldosterone with HDL-C [5, 7–9], while positive associations of aldosterone with triglycerides [7, 9] could not be replicated. As major new findings, our data show that plasma aldosterone is positively associated with LDL-C and non-HDL-C in the general population. As the associations of the aldosterone-to-renin ratio with lipid concentrations were not statistically significant, increasing aldosterone per se seems to control the observed associations.

Our data and the results of the abovementioned studies [5, 7–9, 12] suggest that high aldosterone may have a negative effect on circulating lipid concentrations. This in turn may contribute to the cardiovascular damage observed with inappropriately high aldosterone concentrations [13]. The detrimental effect of aldosterone is most impressively observed in patients with primary aldosteronism, a serious condition with extremely high aldosterone concentrations. Primary aldosteronism causes hypertension, and patients commonly have a high cardiovascular risk [14] and wide range of comorbidities [15]. Yet, a recent study [4] reported that successful treatment of primary aldosteronism resulted in increased serum LDL-C and triglyceride levels as well as decreased HDL-C levels. While these results seem counterintuitive at first sight, the worsening lipid profile under surgical or medical therapy might not result in a higher cardiovascular risk. Instead, the manifold therapy benefits, including a lowering of blood pressure, probably outweigh the adverse lipid changes [15]. In addition, the effects observed in primary aldosteronism may not be representative of the general population. Apart from primary aldosteronism, negative effects of increasing aldosterone have been reported in several studies, for example, in patients with chronic heart failure [16], myocardial infarction [17], or the general population [18]. In these studies [16–18], increasing aldosterone was related to an elevated cardiovascular mortality [17, 18] or incident hypertension [16]. Whether our findings of increasing LDL-C and non-HDL-C and decreasing HDL-C with increasing aldosterone contribute to incident cardiovascular events in the general population and whether a lowering of circulating aldosterone concentrations may be cardioprotective cannot be answered by our cross-sectional study. However, previous studies point into that direction [19, 20]. Thus, a combined RAAS blockade with an angiotensin-converting enzyme inhibitor and an angiotensin II AT1 receptor blocker led to increased HDL-C and decreased LDL-C levels in patients with diabetes and hypertension [20]. Moreover, treatment with mineralocorticoid receptor antagonists in addition to standard therapy improved cardiovascular morbidity and mortality in heart failure patients [19].

The mechanisms underlying the associations between circulating aldosterone and lipid concentrations are currently poorly understood, but a brought range of pathophysiological explanations has been discussed. The most evident approach relates to the link between obesity and RAAS activity. Adipose tissue, via secretion of factors such as leptin, stimulates adrenal aldosterone secretion [21]. Aldosterone, via the mineralocorticoid receptor, may in turn mediate adipocyte differentiation, thereby contributing to adipogenesis and inflammation [22–24]. Indeed, BMI and aldosterone were closely linked in our study. A linear regression model adjusted for age and sex demonstrated a positive relation between the measures (*β-*coefficient = 0.010, standard error = 0.004, *p* < 0.01). Nevertheless, the associations between aldosterone and LDL-C, HDL-C, and non-HDL-C remained significant after adjustment for BMI. Another approach linking aldosterone and lipid concentrations aims at HDL-C, which may directly inhibit adrenal aldosterone secretion by modulating the sensitivity of the adrenal zona glomerulosa [6]. Other studies (reviewed by Trevisan et al. [25]) showed that high aldosterone concentrations may induce kidney injury and proteinuria. The herewith associated loss of albumin, lipoproteins, and liver proteins may affect LDL-C and HDL-C synthesis [26].

The present study stands out due to its large sample size as well as comprehensive and standardized data collection. Limitations arise from the observational study design, which implies that the results of this study cannot be interpreted in a causal way. Our cross-sectional analysis is further not suited to draw conclusions on related incident cardiovascular outcomes. Moreover, data on salt or water intake of the study participants was not collected. While we have no reason to assume that our study participants consume more than average amounts of salt or water, we also cannot exclude that single individuals may do so. Finally, insights into the pathophysiology of the topic cannot be generated by our study.

Taken together, the present data show that plasma aldosterone is positively associated with LDL-C and non-HDL-C in the general population. Furthermore, our study confirms the previously reported inverse association of plasma aldosterone with HDL-C. Our data suggest that aldosterone concentrations within the physiological range may be related to alterations of lipid metabolism.

## Figures and Tables

**Table 1:** Characteristics of the study population.

Characteristics	Quartiles of the plasma aldosterone concentration (PAC)				*p*
	Q1 (*n* = 415)	Q2 (*n* = 435)	Q3 (*n* = 447)	Q4 (*n* = 434)	
Male (%)	45.1	46.4	45.9	45.9	0.98
Age (years)	46.0 (37.0–58.0)	46.0 (37.0–58.0)	45.0 (37.0–57.0)	42.0 (35.0–53.0)	<0.01
BMI (kg/m^2^)	25.7 (23.6–29.1)	25.7 (23.2–28.2)	26.4 (23.6–29.1)	26.2 (23.1–29.8)	0.21
HbA1c (%)	5.20 (4.90–5.50)	5.10 (4.80–5.50)	5.10 (4.80–5.50)	5.10 (4.70–5.40)	0.11
Diabetes mellitus (%)	5.30	4.61	3.80	6.00	0.48
eGFR (ml/min/1.73 m^2^)	89.8 (78.8–103.8)	89.0 (78.6–102.5)	89.1 (76.0–100.8)	89.2 (78.3–99.8)	0.50
Systolic blood pressure (mmHg)	125 (115–137)	127 (114–138)	126 (116–139)	124 (115–137)	0.47
Diastolic blood pressure (mmHg)	79.5 (74.0–86.0)	79.5 (73.5–87.0)	80.5 (75.0–88.0)	80.5 (74.0–88.5)	0.34
Hypertension (%)	25.3	26.0	28.6	27.3	0.70
Intake of oral contraceptives (%)^∗^	13.6	15.9	8.26	14.5	0.07
PAC (ng/l)	17.0 (12.0–21.0)	33.0 (28.0–38.0)	49.0 (43.0–54.0)	78.0 (67.0–96.0)	<0.01
PRC (ng/l)	6.50 (4.50–9.00)	7.60 (4.70–10.70)	8.30 (5.40–11.70)	10.05 (7.30–14.90)	<0.01
ARR	2.43 (1.59–3.57)	4.51 (2.99–6.59)	6.17 (4.10–8.62)	7.90 (5.66–11.04)	<0.01
Potassium (mmol/l)	4.26 (4.08–4.50)	4.30 (4.10–4.50)	4.32 (4.18–4.59)	4.37 (4.13–4.58)	<0.01
Sodium (mmol/l)	139 (138–141)	139 (138–141)	139 (138–141)	139 (138–141)	0.18
Total cholesterol (mmol/l)	5.37 (4.70–6.20)	5.60 (0.97–6.40)	5.51 (4.80–6.24)	5.40 (4.70–6.30)	0.26
LDL cholesterol (mmol/l)	3.35 (2.81–4.04)	3.56 (2.80–4.36)	3.54 (2.86–4.17)	3.42 (2.85–4.20)	0.28
HDL cholesterol (mmol/l)	1.22 (0.95–1.55)	1.20 (0.97–1.50)	1.16 (0.89–1.49)	1.13 (0.90–1.45)	0.04
Non-HDL cholesterol (mmol/l)	4.10 (3.43–4.94)	4.31 (3.42–5.25)	4.33 (3.45–5.10)	4.24 (3.45–5.09)	0.34
Triglycerides (mmol/l)	1.20 (0.83–1.92)	1.30 (0.88–2.03)	1.28 (0.88–1.98)	1.36 (0.88–1.95)	0.29

Data are median (1st–3rd quartile) or proportions. Group differences were tested with chi-squared or Kruskal-Wallis tests. ^∗^Intake of oral contraceptives in women. ARR: aldosterone-to-renin ratio; BMI: body mass index; eGFR: estimated glomerular filtration rate; HDL-C: high-density lipoprotein cholesterol; LDL-C: low-density lipoprotein cholesterol; PRC: plasma renin concentration.
